# Abnormal origin of posterior circumflex humeral artery and subscapular artery: case report and review of the literature

**DOI:** 10.1590/1677-5449.001917

**Published:** 2017

**Authors:** Rajani Singh

**Affiliations:** 1 All India Institute of Medical Siences Rishikesh – AIIMS, Department of Anatomy, Rishikesh, Uttrakhand, India.

**Keywords:** axillary artery, variation, radial nerve, pectoralis minor, artéria axilar, variações, nervo radial, peitoral menor

## Abstract

The subscapular, anterior circumflex, and posterior circumflex arteries arise from the third part of the axillary artery. During dissection of the right upper limb of the cadaver of a 70-year-old male, a common trunk was observed arising from the third part of the axillary artery which, after traveling for 0.5 cm, bifurcated into subscapular and posterior circumflex humeral arteries. The common trunk was crossed anteriorly by the radial nerve. The medial nerve was formed by medial and lateral roots on the medial side of the third part of the axillary artery, remaining medial to the brachial artery up to the cubital fossa and then following its usual course thereafter. Awareness of the vascular variations observed in the present case is important when conducting surgical procedures in the axilla, for radiologists interpreting angiographs, and for anatomy-pathologists studying rare findings.

## Resumo

As artérias subescapular, circunflexa anterior e circunflexa posterior se originam da terceira parte da artéria axilar. Durante a dissecção de membro superior direito de um cadáver humano com 70 anos de idade, do sexo masculino, um tronco comum foi observado originando-se da terceira parte da artéria axilar, após um percurso de 0,5 cm, bifurcando-se em artéria subescapular e artéria circunflexa posterior do húmero. O tronco comum era cruzado anteriormente pelo nervo radial. O nervo medial era formado por raízes medial e lateral, no lado medial da terceira parte da artéria axilar, permanecendo em posição medial à artéria braquial até a fossa cubital e seguindo seu curso usual a partir de então. Conhecimento das variações vasculares observadas neste caso é importante ao executar procedimentos cirúrgicos na axila, para radiologistas que interpretam radiografias, e também para anatomo-patologistas que estudam achados raros.

## INTRODUCTION

The axillary artery is a continuation of the subclavian artery and begins at the outer border of the first rib. The axillary artery is anatomically divided into three parts with relation to the pectoralis minor muscle. The first part lies proximal, the second part deep, and the third part distal to the pectoralis minor muscle.^1^ The first part of the artery gives rise to the superior thoracic artery. The second part of the axillary artery gives off the lateral thoracic and thoracoacromial arteries. The subscapular, anterior, and posterior circumflex humeral arteries originate from the third part of axillary artery.^2^ Although variations in the branching pattern of the axillary artery are common, a rare anatomical variation of the origin of the subscapular and posterior circumflex humeral arteries from the axillary artery combined with an anomalous relationship between the radial nerve and the common trunk and a variant formation and course of the median nerve all detected simultaneously nevertheless merit reporting. Besides its importance as an anatomical curiosity, knowledge of these variations relating to variant configurations of the axillary, subscapular, and posterior circumflex humeral arteries and the radial and median nerves is clinically important for orthopedic and vascular surgeons to avoid complications during various surgical procedures in the axilla. Additionally the neural variations may be useful when conducting regional neurosurgery and for successful repair of peripheral nerves. Hence the case is reported here.

## CASE REPORT

**Figure 1:**
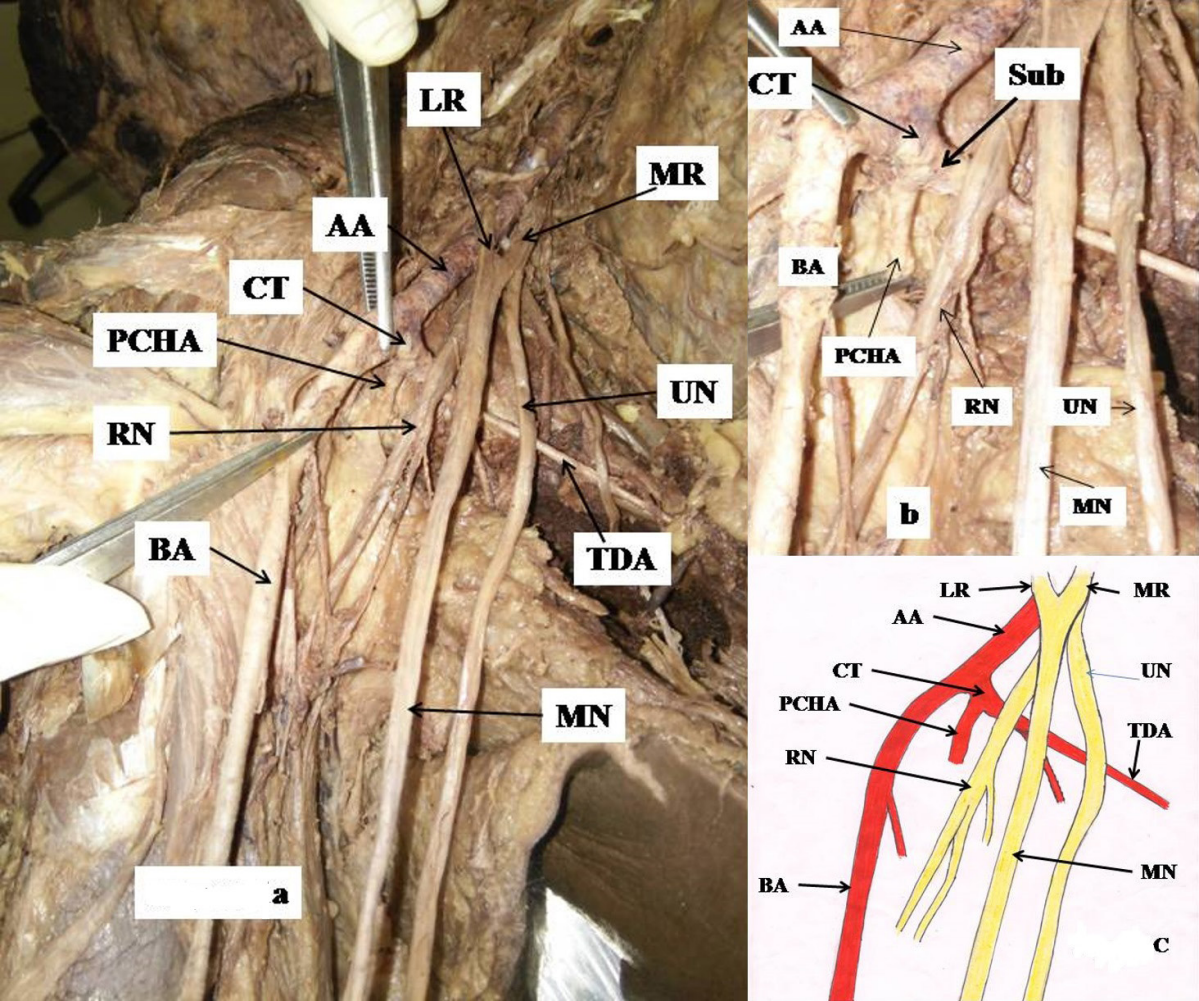
(a) showing a common trunk from the third part of the axillary artery and abnormal positions of the radial nerve and median nerve; (b) enlarged view; (c) schematic diagram showing variations of the third part of the axillary artery and abnormal disposition of the radial nerve. AA: axillary artery; BA: brachial artery; CT: common trunk; LR: lateral root; MN: median nerve; MR: medial root; PCHA: posterior circumflex humeral artery; RN: radial nerve; Sub: subscapular artery; TDA: thoracodorsal artery; UN: ulnar nerve.

During dissection of the right upper limb of the cadaver of a 70-year-old male fixed in 10% formaline, conducted at the department of anatomy, AIIMS Rishikesh, Uttrakhand, India, multiple variations were observed in the axilla. The third part of the axillary artery gave off an anomalous common trunk, which, after traveling 0.5 cm, bifurcated into subscapular and posterior circumflex femoral arteries (Figure 1). The common trunk was crossed anteriorly by the radial nerve. The lateral root from the lateral cord crossed the axillary artery and fused with the medial root of the medial cord, forming the median nerve, lying medial to the axillary artery. This remained medial to the brachial artery throughout the arm and cubital fossa. There was no abnormality in the left upper limb.

## DISCUSSION

**Figure 2:**
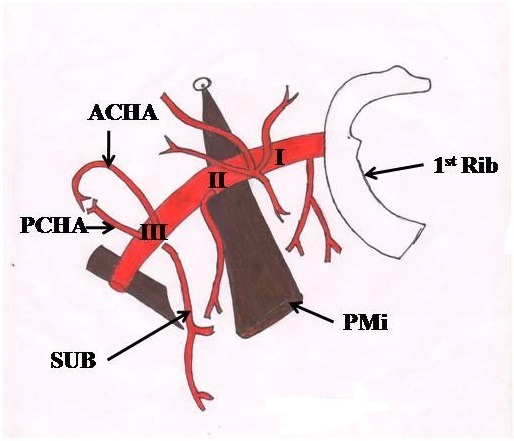
Showing the branches of the axillary artery as described in standard anatomy text books. I, II and III represent the three parts of the axillary artery. ACHA: anterior circumflex humeral artery; PCHA: posterior circumflex humeral artery; PMi: pectoralis minor; Sub: subscapular artery.

As described in standard anatomy text books, the anterior circumflex, posterior circumflex humeral arteries, and, subsequently, the subscapular artery normally emanate from the third part of the axillary artery (Figure 2). A common trunk arising from the third part of the axillary artery and bifurcating into posterior circumflex humeral and subscapular arteries has been reported by Gaur et al.,^3^ similar to the present study, but our case is different from that described by Gaur et al. as regards the anomalous configuration of the radial nerve with respect to the common trunk. Normally, the radial nerve lies deep to the 3rd part of the axillary artery and branches. In the present case, it crossed the common trunk anteriorly, from medial to lateral. The radial nerve may compress the common trunk, causing ischemic changes in the area supplied by the posterior circumflex humeral and subscapular arteries. Moreover, the median nerve, which usually remains lateral to the axillary artery, is located medial to it. The median nerve continued anomalously, medial to the brachial artery up to the cubital fossa. This is the first time that formation of a common trunk and its bifurcation into posterior circumflex and subscapular arteries in combination with abnormal disposition of the radial nerve has been detected and similar variant configurations of the median nerve have rarely been described in the literature.

Bergman et al.^4^ reported emergence of anterior circumflex humeral, posterior circumflex humeral, subscapular, and profunda brachii arteries from a common trunk arising from the third part of the axillary artery. Saeed et al.^5^ reported the origin of a common subscapular-circumflex humeral trunk from the third part of axillary artery, trifurcating into subscapular, anterior circumflex humeral, and posterior circumflex humeral arteries in 3.8% of cases. Ramesh et al.^6^ described the emergence of a common trunk from the third part of the left axillary artery. This common trunk divided into subscapular, anterior circumflex humeral, posterior circumflex humeral, profunda brachii, and ulnar collateral arteries. Vijaya et al.^7^ reported a common trunk that emerged from the third part of the axillary artery and then divided into anterior circumflex humeral, posterior circumflex humeral, subscapular, radial collateral, middle collateral, and superior ulnar collateral arteries. Here, the profunda brachii artery was absent. Astik and Dave^8^ found a common trunk from the third part of the axillary artery in 25% of a sample of 40 limbs. Of these, in 10% the common trunk gave origin to anterior circumflex humeral, posterior circumflex humeral, subscapular, and profunda brachii arteries, whereas in 15% of these cases anterior circumflex humeral, posterior circumflex humeral, and profunda brachii arteries originated from the common trunk.

### Embryological background

The arteries of the limbs arise as a number of vessels contributing to a primitive capillary plexus, but eventually only one trunk persists – the subclavian artery – with the positions and relations of the seventh intersegmental artery, probably representing its lateral branch. The main trunk to the upper limb, the axillary, forms later. The anomalous blood vessels observed in the present case could be due to^9^ (i) choice of unusual paths in the primitive vascular plexuses, (ii) persistence of vessels normally obliterated, (iii) disappearance of vessels normally retained, (iv) incomplete development, and (v) fusions or absorption of parts that are usually distinct.

Awareness of variations in the branching pattern of the axillary artery is essential when performing bypasses between the axillary and subclavian arteries in surgical treatment of subclavian artery occlusions.^10^ Aneurysms and axillary artery traumas may require reconstructive interventions. The variations highlighted in the present case may present difficulties in this type of procedure. Aneurysms of the axillary artery and its branches are often observed in baseball pitchers.^11^ Repetitive positional compression of the axillary artery in athletes can cause focal intimal hyperplasia, aneurysm formation, segmental dissection, and branch vessel aneurysms. These conditions favor thrombosis and distal embolism and may need positional arteriography for diagnosis.^12^ Variant configuration of branches of the axillary artery similar to the present case may increase positional compression of the common trunk, due to its variant relation to the radial nerve, and so increase propensity to the aforementioned pathologies. The axillary arteries are used as cannulation sites in cardiopulmonary bypass and thoracic and aortic procedures, and for insertion of intra-aortic balloon pumps. They are also under consideration for use as inflow vessels in coronary artery surgery.^13^ Variant common trunks from the axillary artery, as observed in the current study, can be considered for cannulation. Radiological studies can thus be performed before proceeding to the aforementioned procedures. It has been shown that injection of oily suspension into deltoid muscle can cause ischemic changes in the scapular and pectoral region. It was presumed that these changes were caused by action of medication on nerves or entrance of substance into blood vessels causing embolism.^14^ In the present case, the radial nerve crossed the common trunk, which may compress the common trunk emanating from the axillary artery and cause ischemia of structures supplied by branches of the common trunk. If one is unaware of this variation, the radial nerve could be injured during surgical procedures in the axilla.

Awareness of the variations observed in the present case may therefore be essential for vascular surgeons during surgical procedures in the axilla and for radiologists, to prevent misinterpretation of angiographs, and of interest to anatomists studying rare variations.

## References

[1] Standring S (2008). Gray’s Anatomy: the anatomical basis of clinical practice.

[2] Snell R (2004). Clinical anatomy for medical students.

[3] Gaur S, Katariya SK, Vaishnani H (2012). A cadaveric study of branching pattern of the axillary artery. Int J Biol Med Res..

[4] Bergman RA, Thompson SA, Afifi AK, Saadeh FA (1988). Compendium of human anatomic variations.

[5] Saeed M, Rufai AA, Elsayed SE, Sadiq MS (2002). Variations in the subclavian-axillary arterial system. Saudi Med J.

[6] Ramesh RT, Shetty P, Suresh R (2008). Abnormal branching pattern of the axillary artery and its clinical significance. Int J Morphol.

[7] Vijaya PS, Venkata RV, Satheesha N, Mohandas R, Sreenivasa RB, Narendra P (2006). A rare variation in the branching pattern of the axillary artery. Indian J Plast Surg.

[8] Rajesh A, Urvi D (2012). Variations in branching pattern of the axillary artery: a study in 40 human cadavers. J Vasc Bras.

[9] Arey LB (1957). In development of the arteries, developmental anatomy.

[10] Bhat KMR, Gowda S, Potu BK, Rao MS (2008). A unique branching pattern of the axillary artery in a South Indian male cadaver. Bratisl Med J.

[11] Schneider K, Kasparyan NG, Altchek DW, Fantini GA, Weiland AJ (1999). An aneurysm involving the axillary artery and its branch vessels in a major league baseball pitcher: a case report and review of the literature. Am J Sports Med.

[12] Duwayri YM, Emery VB, Driskill MR (2011). Positional compression of the axillary artery causing upper extremity thrombosis and embolism in the elite overhead throwing athlete. J Vasc Surg.

[13] Karambelkar RR, Shewale AD, Umarji BN (2011). Variations in branching pattern of axillary artery and its clinical significance. Anatomica Karnataka..

[14] Duque FL, Chagas CA (2009). Intramuscular accident with drug injection in the deltoid muscle: local and distant lesions, review of 32 cases. J Vasc Bras.

